# Primary bone lymphoma: report of a case with multifocal skeletal involvement

**DOI:** 10.2349/biij.3.4.e52

**Published:** 2007-10-01

**Authors:** K Rahmat, ML Wastie, BJJ Abdullah

**Affiliations:** Department of Biomedical Imaging, Faculty of Medicine, University of Malaya, Kuala Lumpur, Malaysia

**Keywords:** Lymphoma, bone, anaplastic large cell lymphoma, radionuclide scintigraphy

## Abstract

Primary bone lymphoma is an uncommon tumour accounting for approximately 4-5% of extra nodal lymphoma and less than 1% of all Non-Hodgkin’s lymphoma. The radiographic appearance of primary bone lymphoma is variable. As lesions frequently resemble other disease processes namely chronic osteomyelitis and metastases, further imaging evaluation and histopathological examination allow early identification for appropriate treatment. The authors present a case of anaplastic large cell lymphoma of bone presenting with multifocal osseus involvement.

## INTRODUCTION

Primary lymphoma of bone (PLB), which is defined as lymphoma localised to the bone without evidence of lymph nodes or other tissues at presentation, is one of the rarest primary bone malignancies, accounting for less than 5% of all primary bone tumours [1]. The vast majority of bone lymphomas are of the Non-Hodgkin’s type, with Hodgkin’s disease and Burkitt’s lymphoma accounting for the rest.

The radiographic appearances of PLB are variable and because the lesions may not be obvious on plain radiography, other modalities such as radionuclide bone scintigraphy or magnetic resonance imaging (MRI) should be utilised. Primary bone lymphoma has a better prognosis than many other malignant tumours, henceforth early identification allows for appropriate treatment. The authors present a young adult with primary bone lymphoma i.e., the anaplastic large cell type with its interesting clinical, radiological and radionuclide correlation.

## CASE REPORT

A 26-year-old male presented with persistent pain and swelling of the left jaw for the past two months following a left 1st molar tooth extraction. There was an associated history of prolonged pyrexia associated with chills and rigors. Patient was treated with a course of antibiotics as infection of the extraction site was suspected. Several weeks later, he presented to an oromaxillofacial surgeon as the symptoms progressed. Physical examination revealed an ill-defined hard swelling arising from the body of the mandible with an exuberant soft tissue growth anterior to the left last molar. Full blood count showed hemoglobin of 127 g/L, white blood cell 11.7 x 10^9^ and platelets 397 x 10^9^. The ESR was 48 mm/hour, which was mildly elevated. He was not known to be immunocompromised.

**Figure 1:**
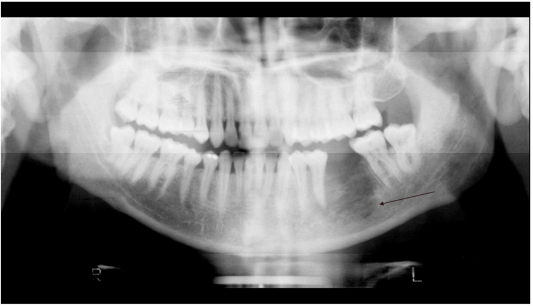
Orthopantomogram shows lytic destruction of the left side involving the left first molar bed (arrow) and the root of the 2nd premolar.

**Figure 2:**
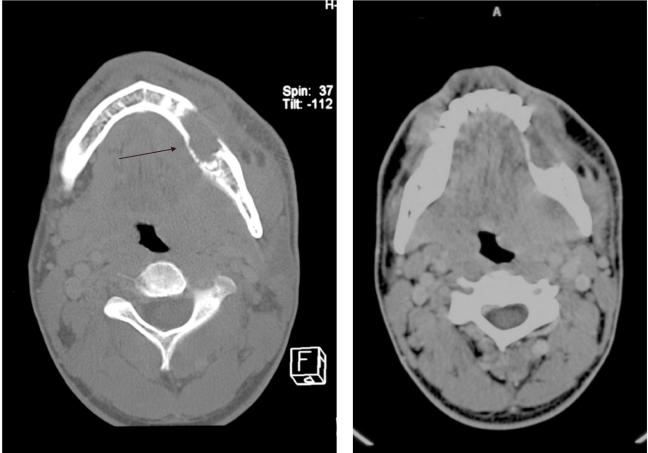
CT scan at level of body of the mandible (left: bone WW: 2344, WC: 440 and right: soft tissue window WW: 250, WC: 50) showing lytic destruction (arrow in left figure) with associated soft tissue swelling. Enlarged bilateral anterior cervical nodes noted.

An orthopantomogram (OPG) (Figure 1) and a contrast enhanced computed tomography (CT) scan of the mandible showed lytic destruction in the body of the mandible, and anterior and lateral alveolar process associated with a soft tissue mass (Figure 2). Numerous cervical and submental lymphadenopathy was noted. Excisional tissue biopsy and, culture and sensitivity of the soft tissue as well as the mandible showed a heavy growth of *Streptococcus oralis, *and *Enterococcus* within chronic granulation tissue. The impression at that time was of chronic osteomyelitis of the mandible. He subsequently developed pain in the occipital region and thoracic spine. However, plain radiographs of these sites were normal.

**Figure 3:**
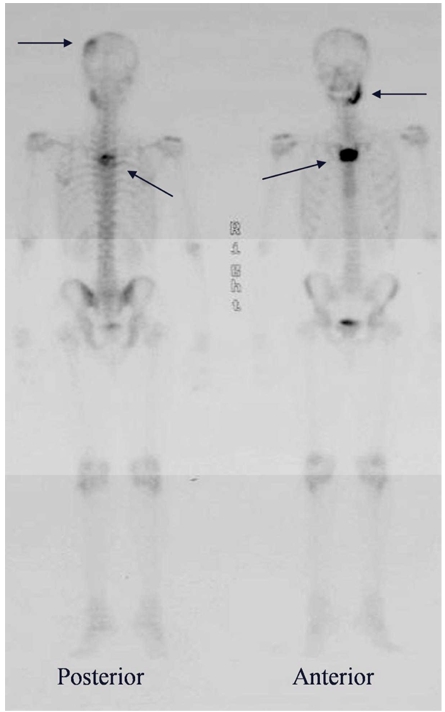
Radionuclide bone scintigraphy using MDP 99m-Technicium showing intense radiotracer uptake in the left side of mandible, manubrium sterni, upper thoracic vertebra and the skull vault (arrows).

In view of the poor response to various courses of antibiotic treatment and progressive bone pain, radionuclide bone scintigraphy (99mTC MDP) was performed. This revealed intense radiotracer uptake in multiple sites in the mandible, manubrium sterni , the upper thoracic and the skull vault (Figure 3). At this point, a differential diagnoses of multifocal metastases, Langerhans Histiocytosis and skeletal lymphoma was made. CT scan of the thorax and abdomen showed no internal organ involvement or abdominal lymphadenopathy.

**Figure 4:**
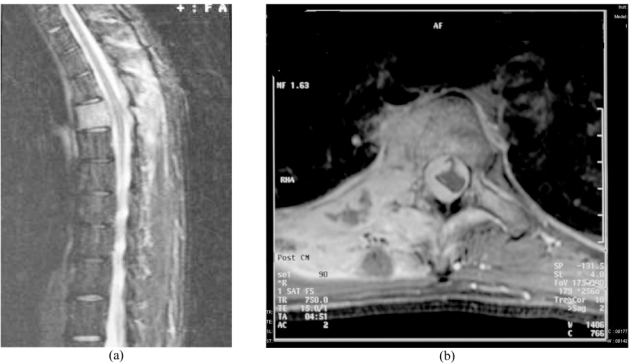
a) Sagittal MRI (STIR, TR 3586.0 TE 60.0) showed abnormally increased signal intensity in the T4 vertebral body and the subcutaneous tissues of the thoracic paravertebral region; b) Axial Post gadolinium image revealed heterogenous enhancement of the vertebral body, right pedicle, laminae, and paravertebral soft tissues at the T4 vertebral level.

MRI of the spine showed abnormally high signal changes of T3 to T5 paravertebral region, erector spinae and trapezius muscle on short time inversion recovery (STIR) images. Axial post-gadolinium image revealed heterogenous enhancement of the vertebral body, right pedicle, laminae, and paravertebral soft tissues at the T4 vertebral level (Figure 4). There was associated intraspinal extradural infiltration with indentation of the spinal cord at the T4 vertebral level.

The patient then proceeded to surgery. Intraoperatively, there was extensive tumour involving the right paraspinal muscles adherent to the dural sac. Posterior decompression and fixation with instrumentation was performed. Histopathology showed anaplastic large cell lymphoma. He was started on a chemotherapy regimen. Follow-up CT scan done in the middle of the chemotherapy cycle showed regression of the paravertebral lesions as well as the soft tissue mass at the left mandibular region. He is currently on follow-up at the Hematology Clinic.

## DISCUSSION

Primary bone lymphoma (PBL) is rare accounting for approximately 4-5% of extra nodal lymphoma and less than 1% of all Non-Hodgkin’s lymphoma [2]. The criteria for a diagnosis of PBL initially suggested by Coley in 1950 with minor modifications are as follows: ‘Lymphoma presenting in an osseous site with no evidence of disease elsewhere for at least six months after diagnosis’. The presence of regional lymph node involvement does not exclude a diagnosis of PBL, but a histological examination of the lymph node is necessary. Although the original criteria of Coley implied the involvement of solitary bone, Ostrowski *et al* [3] subclassify osseous lymphoma into four groups. In their classification, Group 1 consists of solitary primary bone lymphoma and Group 2 encompasses cases in which more than one bone is affected but no nodal or visceral disease is present. Group 3 includes cases with distant nodal disease and Group 4 with visceral disease.

Anaplastic large cell lymphoma (ALCL) represents approximately 2% of all Non-Hodgkin’s lymphomas according to the recent Non-Hodgkin’s Lymphoma Classification Project [4]. ALCL commonly involves nodal as well as a wide variety of extranodal sites, although primary or secondary involvement of bone is rare [5]. Anaplastic large cell lymphoma (ALCL), previously known as Ki-1 lymphoma, is a heterogeneous group of Non-Hodgkin’s lymphomas of T-cell, B-cell, and null-cell phenotypes. Subsequently, it was recognised that T-cell and null-cell ALCL occur more often in younger patients and generally have a better prognosis than histologically similar B-cell neoplasms [1].

PBL occurs in patients from 1 year 6 months to 86 years (median range 36-56 years) with a peak prevalence among patients in the 6th and 7th decade of life. The femur (29%) is the most common site of predilection followed by (in descending order of frequency): pelvis (19%), humerus (13%), skull (11%) and tibia (10%) [6]. Vertebral involvement is not unusual. In the long bones, lymphoma is usually metaphyseal or diaphyseal in location, rarely involving the epiphysis. When primary bone lymphoma disseminates, its spread to other bony sites is recognised [7].

The clinical presentation reflects the nature of the disease process, usually manifesting as insidious and intermittent bone pain that may persist for months. Other signs include local swelling, palpable mass and systemic symptoms such as pyrexia and loss of weight, all the manifestations that this patient had. But these symptoms are also associated with that of chronic osteomyelitis. Plain radiographic appearance is variable and non-specific. Lymphoma of bone almost invariably arises in the medullary portion of the bone presenting as predominantly destructive and osteolytic bony lesions. The lytic pattern may be permeative characterised by numerous small, elongated rare fractions that are parallel to the long axis of the bone and relatively uniform in size or ‘moth-eaten’ – a pattern of many medium to large areas of radiolucency, which are poorly marginated. Occasionally, the disease may manifest with focal lytic areas with well-defined margins.

The lytic destructive pattern is the most common radiographic appearance of PBL and it was reported in approximately 70% of 237 cases reviewed by Mulligan *et al* [1]. Cortical breakthrough, pathological fracture and soft tissue masses represent a more aggressive pattern of involvement and poor prognosis. Periosteal reactions and presence of sequestra are recognised associated imaging findings. Other plain radiographic findings such as blastic-sclerotic pattern and near absence of detectable abnormalities have been described. Cases such as these are best further evaluated by MRI and bone scintigraphy, which clearly demonstrate patterns of abnormal marrow replacement.

The appearances of primary bone lymphoma on radionuclide bone imaging are usually non specific, depicting lesions of abnormality as areas of increased tracer uptake. This pattern may also be seen with other marrow infiltrating tumours such as leukemia, multiple myeloma, metastatic disease as well as multifocal osteomyelitis. In view of this patient’s young age and the combination of scintigraphic abnormality in the mandible, skull and sternum, metastatic disease was thought unlikely and a differential diagnosis of skeletal lymphoma was suggested.

MRI typically shows a focus of marrow replacement and the extent of surrounding soft tissue mass. Generally, lesions demonstrate low signal intensity on T1-weighted imaging within the marrow with bright signal intensity on T2-weighted imaging [8]. However presence of fibrosis may show low signal intensity on both sequences [9]. STIR also delineate the abnormality in the bone marrow. Gadolinium contrast enhanced images will demonstrate areas of enhancement within the lesion. MRI is also particularly useful in evaluation of spine involvement and any spinal cord compression.

This particular case demonstrated the clinicians’ as well as the radiologists’ initial diagnostic difficulty. This may be attributed to inadequate tissue sampling especially if it was not done under imaging guidance. One solution to overcome this difficulty is to perform an open bone biopsy, which this patient had during posterior spinal decompression surgery. Adequate biopsy sample for histopathology, immunotyping and immunohistochemistry study of the lymphoma cells is critical in the definitive diagnosis of ALCL.

Primary lymphoma of bone usually responds well to a combination of radiation therapy and chemotherapy regimens with an overall response rate of 94% in some series and a 5-year survival rate, better than that achieved with most other primary osseous malignancy [10]. ALCL is a high-grade lymphoma and it usually needs prompt treatment with chemotherapy. The current treatment approach for primary systemic ALCL is identical to that for other types of diffuse aggressive Non-Hodgkin’s Lymphomas. A combination of cyclophosphamide, doxorubicin, vincristine, and prednisone (CHOP) is the standard first-line treatment. If CD20 antigens are positive, rituximab should be added. Radiation therapy to bulky sites of disease may be necessary after completion of chemotherapy. From the clinical and radiological point of view, lymphoma of bone should be considered in the differential diagnosis of a young adult presenting with multifocal osseous involvement.
